# The structural changes of gray matter in Parkinson disease patients with mild cognitive impairments

**DOI:** 10.1371/journal.pone.0269787

**Published:** 2022-07-20

**Authors:** Lihua Li, Bingjun Ji, Ting Zhao, Xuan Cui, Jingtao Chen, Zhenyu Wang

**Affiliations:** 1 School of Education, Xinyang College, Xinyang, China; 2 Department of Radiology, Sunshine Union Hospital, Weifang, China; 3 Department of Neurology, Sunshine Union Hospital, Weifang, China; 4 Department of Medical Imaging, Weifang Medical University, Weifang, China; IRCCS Foundation Stella Maris: IRCCS Fondazione Stella Maris, ITALY

## Abstract

**Objectives:**

Parkinson disease (PD) is associated with cognitive impairments. However, the underlying neural mechanism of cognitive impairments in PD is still not clear. This study aimed to investigate the anatomic alternations of gray matter in PD patients with mild cognitive impairment (MCI) and their associations with neurocognitive measurements.

**Methods:**

T1-weighted magnetic resonance imaging (MRI) data were acquired from 23 PD patients with MCI, 23 PD patients without MCI, and 23 matched healthy controls. The MRI data were analyzed using voxel-based morphometry (VBM) and surfaced-based morphometry (SBM) methods to assess the structural changes in gray matter volume and cortical thickness respectively. Receiver operating characteristic (ROC) analysis was used to examine the diagnostic accuracies of the indexes of interest. The correlations between the structural metrics and neurocognitive assessments (e.g., Montreal cognitive assessment, MOCA; Mini-mental state examination, MMSE) were further examined.

**Results:**

PD patients with MCI showed reduced gray matter volume (GMV) in the frontal cortex (e.g., right inferior frontal gyrus and middle frontal gyrus) and extended to insula as well as cerebellum compared with the healthy controls and PD patients without MIC. Thinner of cortical thickens in the temporal lobe (e.g., left middle temporal gyrus and right superior temporal gyrus) extending to parietal cortex (e.g., precuneus) were found in the PD patients with MCI relative to the healthy controls and PD patients without MCI.ROC analysis indicated that the area under the ROC curve (AUC) values in the frontal, temporal, and subcortical structures (e.g., insula and cerebellum) could differentiate the PD patients with MCI and without MCI and healthy controls. Furthermore, GMV of the right middle frontal gyrus and cortical thickness of the right superior temporal gyrus were correlated with neurocognitive dysfunctions (e.g., MOCA and MMSE) in PD patients with MCI.

**Conclusion:**

This study provided further evidence that PD with MCI was associated with structural alternations of brain. Morphometric analysis focusing on the cortical and subcortical regions could be biomarkers of cognitive impairments in PD patients.

## 1. Introduction

Parkinson’s disease (PD) is one of the most common neurodegenerative diseases that is characterized by using the progressive loss of dopaminergic neurons in the substantia nigra [1]. The typical clinical hallmarks of PD are not only present motor symptoms (e.g., tremor, rigidity, and postural instability) [2], but also non-motor symptoms (e.g., depression, constipation, and olfactory deficit) [3]. Cognitive impairments are common non-motor symptoms in patients with PD and frequently occur in the disease even prior to diagnosing the disease [4, 5]. PD with mild cognitive impairments (MCI) is widely considered to be a higher risk of developing dementia [6], which can arise in up to 80% of PD patients through the long term [7] and can severely impact on the quality of life and increase the disability of PD patients [8]. Identifying biomarkers of PD with MCI not only contribute to diagnosis, but also to disease progression by early therapeutic intervention. However, the underlying brain pathology leading to cognitive impairments in PD remain largely unknown.

Neuroimaging has provided a promising approach to investigate the various risk factor and identify the brain structural features of PD patients with MCI, which would provide more insights to prevailing theories [9, 10]. In this regard, structural magnetic resonance imaging (sMRI) has been widely applied to be a viable platform for identifying biomarkers of PD. More recently, investigators have used voxel-based morphometry (VBM) to investigate the morphometrics of the brain in PD patients. VBM is a voxel-wise imaging processing method of sMRI that allows detecting subtle morphological changes of gray matter across the whole brain and can quantify alterations in gray matter volume (GMV) in neurological disorders (e.g., PD) [11]. Using the VBM analysis, several studies have investigated neuroanatomical alternations in PD and cognitive impairments, showing widespread atrophy of GMV in the frontal, parietal, and temporal cortices [12–14], and subcortical structures, including the insula, cingulate gyrus, hippocampal gyrus, and cerebellum [12, 13, 15]. These results indicated that the implication of the fronto-temporo-limbic regions could be the main feature of cognitive impairments and could be potential biomarkers for in PD with MCI.

However, only VBM analyses are possible not enough to detect early cortical changes in PD since it only detects voxels for which a specific predicted effect has less sensitivity for overlapping areas [16]. Surface-based morphometry (SBM) analysis can provide powerful tool for estimating cortical thickness of human cerebral cortex from sMRI data [17]. The cortical thickness measurement by SBM is a more direct index of cortical morphology that is less susceptible to registration errors across different brains [18] and is more sensitive to identify regional gray matter changes associated with PD. Several studies have described the cortical atrophy profile in PD by using SBM method. In a small sample, investigators have found that PD patients with MCI showed decreased cortical thickness in the right fronto-parietal regions and left temporo-occipital regions compared to PD patients without MCI [19]. In a relatively large sample, investigators have reported that reduced cortical thickness mainly located in the parietal and temporal regions in PD patients with MCI compared to PD patients without MCI [20]. Other researchers have made similar findings [21]. In addition, neurotransmitter deficits, including dopamine, acetylcholine, and norepinephrine systems [22–24], have been implicated in PD. Brain regions rely on neuromodulation from the neurotransmitter systems to preserve normal function [25]. The degeneration of neurotransmitters has been widely detected in cortical and subcortical areas of PD patients [26] and suggested that the structural abnormalities of brain in PD are associated with the neurotransmitter deficits.

Multimodal imaging approach could be useful for detecting sensitive alternations in PD patients with MCI. Specifically, VBM and SBM approaches seem to provide complementary information about structural changes, as they detect differences of gray matter at the voxel or volume level. The present study used these methods in conjunction to capture complementary aspects of brain pathological alternations in PD patients with MCI and to determine (1) whether different anatomical profiles exist that involve in gray matter patter of brain atrophy; (2) whether the different patterns of structural changes of brain differentiate the patients from the different subtypes and healthy controls; and (3) whether these brain structural measurements are associated with clinical and neurocognitive profiles. We hypothesized that multimodal information would allow us to gather additional knowledge on the characteristics of neurodegeneration of PD with MCI.

## 2. Methods

### 2.1 Participants

The sample included 23 patients clinically diagnosed as PD with MCI and 23 patients diagnosed as PD without MCI. All participants in this study were recruited from the Department of Neurology, Sunshine Union Hospital (Weifang, China) between January 2019 and December 2020. The diagnosis of PD was clinically determined according to the UK Parkinson’s Disease Brain Bank criteria [27]. PD patients without MCI exhibit no impairments on cognitive abilities on any perception [28]. MCI was diagnosed according to the Movement Disorder Society (MDS) Task Force, level I criteria [28]: (1) in the circumstance of established PD, a gradual decrease in cognitive ability reported by either informant or the patient or observed by the clinician; (2) cognitive deficits that were insufficient to interfere substantially with functional independence; (3) Montreal Cognitive Assessment (MoCA) scores <26, but related dementia do not reach clinical criteria [29]. Besides, 23 healthy volunteers matched with age, sex, and education were recruited as healthy control group. Health controls had no cognitive complaints and normal cognition and neurologic examinations. All patients were taking antiparkinsonian drugs including different combinations of L-dopa, catechol-O-methyltransferase inhibitors, and monoamine oxidase inhibitors. To standardize the doses, the L-dopa equivalent daily dose (LEDD) was calculated to express dose intensity of different antiparkinsonian drug [30].

Participants were excluded if they met the following criteria: (1) brain lesion contraindication on MRI; (2) severe concomitant diseases that might influence brain metabolic alterations; (3) history of current psychiatric illness; (4) diagnosed dementia; and (5) moderate-to-severe head rest tremor; and (6) secondary Parkinsonism and Parkinson-plus syndrome. All diagnosis was managed by at least two professional neurologists. This study protocol was approved by the Medical Ethics Committee of the Weifang Medical University and met the Declaration of Helsinki. Written informed consent was obtained from all participants prior to participation in this study.

### 2.2 Clinical and neurocognitive measurements

The demographic data of all participants including age, gender, and education were collected. The clinical data included disease duration of PD, the Unified Parkinson’s Disease Rating Scale (UPDRS_III) score, and the modified Hoehn and Lahr (H&Y) score [31]. Moreover, the cognitive function status was assessed by the Mini-Mental Status Examination (MMSE) [32] in each participant. Montreal Cognitive Assessment (MoCA) [33] were further evaluated in PD patients and five cognitive domains were examined, including attention, memory, visuospatial function, language, and executive function. In addition, we collected the data on the patients’ experienced seizures from clinical records. The demographic and clinical details of all participants are showed in Table 1.

**Table 1:** Demographic, clinical, and neurocognitive characteristics among the three groups.

Variable	Patients with MCI (n = 23)	Patients without MCI (n = 23)	Healthy Controls (n = 23)	P (P1, P2, P3)
Gender (female/male)	11/12	9/14	13/10	0.498
				(0.555,0.237,0.552)
Age (years)	64.30±3.68	63.65±3.63	62.04±3.81	0.111
				(0.056,0.149,0.548)
Education (years)	6.65±1.53	7.43±1.59	7.39±1.47	0.157
				(0.101,0.923,0.095)
Duration (years)	6.30±2.46	6.95±1.87	-	0.316
MOCA	22.96±3.07	26.69±2.12	27.65±1.19	<0.001
				(<0.001, <0.001, <0.001)
MMSE	24.13±1.87	27.43±1.08	28.65±0.94	<0.001
				(<0.001, <0.001, <0.001)
Executive function	1.57±1.20	2.52±0.90	3.22±0.85	<0.001
				(<0.001, <0.05, <0.01)
Attention	3.35±1.90	4.44±1.59	5.83±1.64	<0.001
				(<0.001, <0.05, <0.05)
Memory	1.74±0.81	2.39±1.03	3.52±0.85	<0.001
				(<0.001, <0.001, <0.05)
Visuospatial ability	1.41±1.18	2.46±1.02	3.17±0.94	<0.001
				(<0.001, <0.001, <0.001)
Langue	1.82±1.10	2.88±1.123	3.87±0.97	<0.001
				(<0.001, <0.05, <0.05)
Levodopa doses	398.87±11.31	398.96±11.60	-	0.979
UPDRS-III	29.65±3.41	26.39±2.05	-	0.113
H&Y Stage (1,2)	1.5±0.5	1.3±0.5	-	0.380
Experienced seizures	3	1	-	0.295

Notes: UPDRS, Unified Parkinson Disease rating scale; H&Y, Hoehn and Yahr; MoCA, Montreal cognitive assessment. MMSE, mini-mental state examines. P1 (PD patients with MCI vs. healthy controls), P2 (PD patients without MCI vs. healthy controls), P3 (PD patients with MCI vs. PD patients without MCI).

### 2.3 Imaging data acquisition

The MRI scans were acquired using a Siemens 3.0 T imaging system (Siemens, Munich, Germany) equipped with an 8-channel phased array head coil. The scanning protocol included a high-resolution 3- dimensional T1-weighted magnetization-prepared rapid acquisition gradient-echo volume with the following parameters: TR = 530 ms; TE = 3.42 ms; Field of view = 256 × 256 cm; flip angle = 15°; matrix = 256 × 256; 176 interleaved slices with no gap; voxel size = 1.1 × 1.1 × 1.1 mm^3^. Head motion was minimized by using foam pads and by providing reassurance at the beginning of the scans. A quality check to exclude motion artifacts was executed by 2 researchers independently.

### 2.4 Anatomic data analysis

The anatomical images were processed and analyzed using the CAT12 toolbox implemented in Statistical Parametric Mapping (SPM12; www.fil.ion.ucl.ac.uk/spm). CAT12 provides processing pipelines for both VBM and SBM modules, allowing us to perform all analyses with this software package. For the steps of processing and analysis, the parameters used default settings met the standard protocol (http://www.neuro.uni-jena.de/cat12/CAT12-Manual.pdf). This tool has been widely used and validated in morphometric studies in PD [34, 35]. A two-step quality assurance was also included: first, all images were visually inspected for artifacts (prior to preprocessing); secondly, statistical quality control was performed for overall image quality and inter-subject homogeneity after segmentation.

For VBM analysis, the anatomical images were normalized to a standard template by the diffeomorphic anatomical registration through exponentiated lie algebra (DARTEL) algorithm and then segmented into three voxel classes: gray matter, white matter, and cerebrospinal fluid using partial volume segmentation with MAP approach. Then the regional gray matter volume differences were tested using modulated normalized gray matter maps. The abstracted gray matter maps were smoothed utilizing an 8 mm full width half maximum (FWHM) Gaussian kernel and used for further analysis. For SBM analysis, the cortical thickness was estimated using a projection-based distance measure. The vertex-wise cortical thickness measures were resampled and smoothed by a 12 mm FWHM Gaussian kernel.

### 2.5 ROC analysis

Receiver operating characteristic (ROC) curves of brain structure measurements were used to decide the cutoff values that were associated with optimal sensitivity and specificity for distinguishing patients with PD with MCI from without MCI patients and healthy controls. The areas under the ROC curve (AUC) were used to compare the overall diagnostic performance of the indexes in all regions of interest (ROI).

### 2.6 Correlations analysis

The relationships between the brain structural variables and neurocognitive scores in PD patients with MCI were estimated by utilizing Pearson correlation coefficients. Two-tailed P < 0.05 was regarded to show significance.

### 2.7 Statistical analysis

An independent two-sample t-test was used to identify the regions with significant differences in each of both morphometric measures (GMV with VBM and cortical thickness with SBM) between the groups. Age, gender, education, experienced seizures, and ON medication state as covariates were included in the analysis (for VBM analyses, additionally added total intracranial volume as a covariate). P < 0.05 with a false discovery rate (FDR) correction (size > 50) was considered statistically significant.

The demographic, clinical, and neurocognitive data were analyzed using the SPSS 20 Statistics software package (IBM Corporation, New York, EUA). Two-sample t-test was applied to compare disease duration, Levodopa does, and UPDRS-III scores between the two PD patient groups. Differences in age, education, and neurocognitive scores between all groups were analyzed with one-factor analyses of ANOVA following by post-hoc t-test and Bonferroni correction controlling for multiple comparisons. Categorical variables (gender, H &Y scores, and experienced seizures) were compared using chi-squared tests. P < 0.05 of All tests was considered statistically significant.

## 3. Results

### 3.1 Demographic, clinical, and neurocognitive measurements of participants

The demographic and clinical profiles of all participants are exhibited in Table 1. There were no significant differences among the three groups for gender (P = 0.498), age (P = 0.111), education (P = 0.157). PD patients with MCI did not differ with PD patients without MCI in the disease duration (P = 0.316), UPDRS-III (P = 0.113), H &Y stages (P = 0.380), and experienced seizures (P = 0.295). However, MOCA (P < 0.001) and MMSE (P < 0.001) scores of the PD patients with MCI were significantly worse than that of PD patients without MCI and healthy controls, in line with the clinical diagnosis of each subtype.

### 3.2 GMV differences between the groups

**Fig 1:**
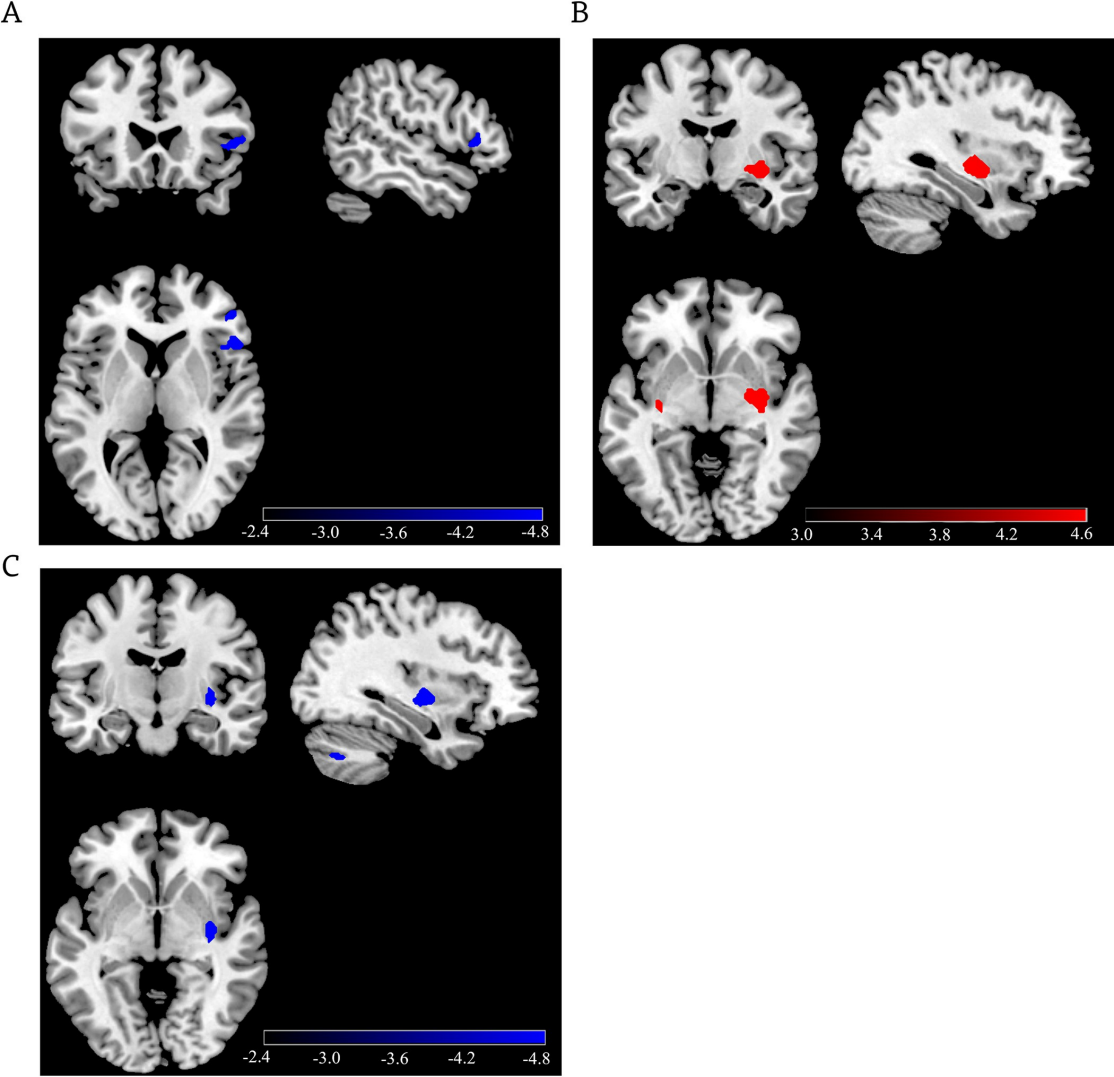
(A) Differences of gray matter volume between PD patients with MCI and healthy controls; (B) Differences of gray matter volume between PD patients without MCI and healthy controls; (C) Differences of gray matter volume between PD patients with MCI and PD patients without MCI. The scale bar shows t values, FDR correction, P < 0.05, size >50.

The patients with PD had extensive structural changes of brain by VBM analysis. Specifically, PD patients with MCI showed reduced GMV in the frontal cortex (e.g., right inferior frontal gyrus and middle frontal gyrus), while PD patients without MCI exhibited increased GMV in the putamen compared to healthy controls (Table 2 and Fig 1). In addition, PD patients with MCI had decreased GMV in the right insula and right cerebellum relative to PD patients without MCI (Table 2 and Fig 1).

**Table 2:** Differences of GMV between the groups by VBM analysis.

Comparisons	Brain regions	MNI Coordinate			t	Voxels
		x	y	z		
PD patients with MCI vs. healthy controls	R Middle Frontal Gyrus	39	54	11	-4.35	83
	R Inferior Frontal Gyrus	51	24	6	-4.27	186
PD patients without MCI vs. healthy controls	R Putman	33	-11	-5	4.37	389
	L Putman	-33	-15	-5	3.76	57
PD patients with MCI vs. PD patients without MCI	R Insula	35	-11	-3	-4.54	176
	R Cerebellum (louble VIII)	30	-65	-42	-3.74	121

Notes: Left; R, right.

### 3.3 Cortical thickness differences between the groups

**Fig 2:**
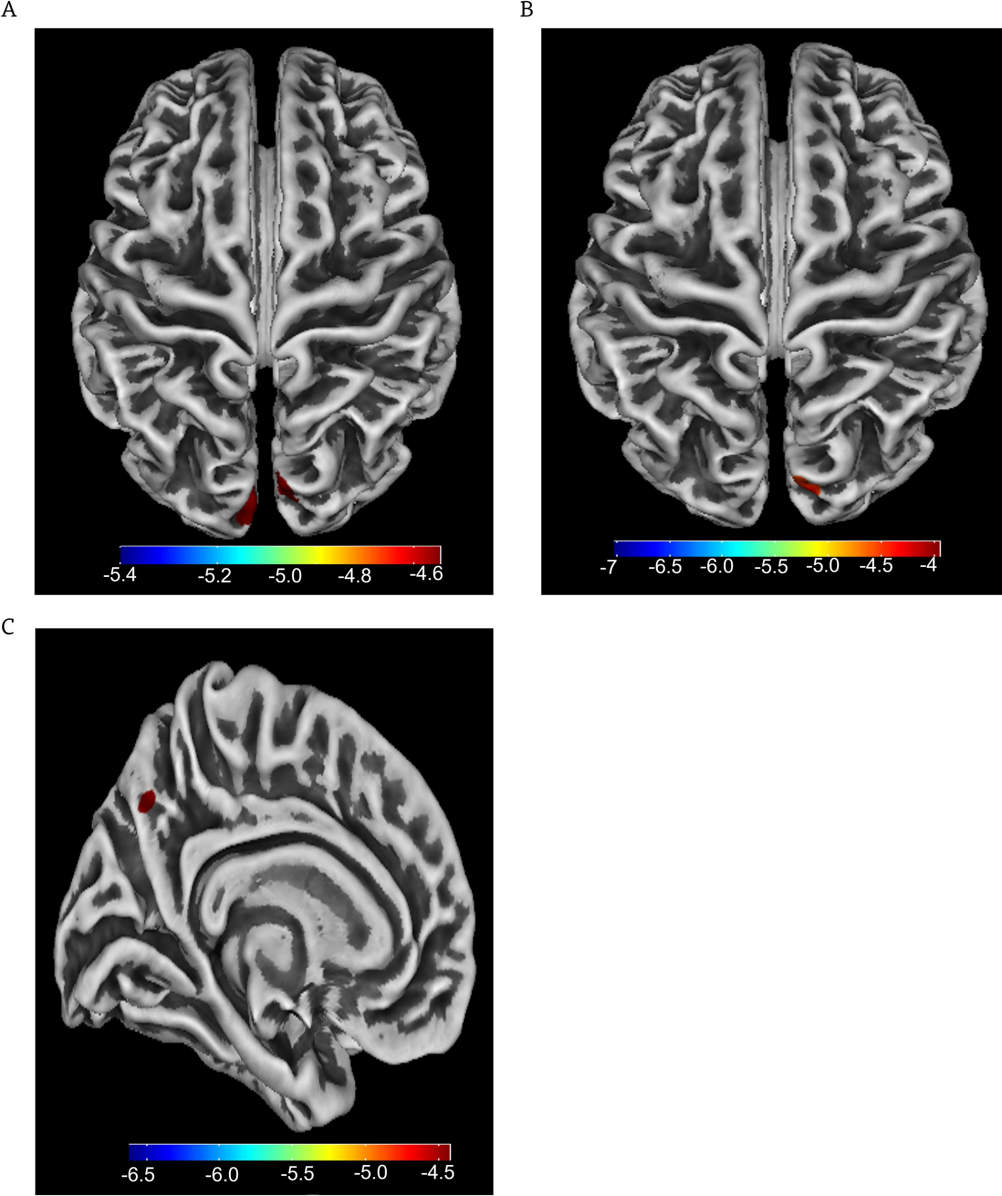
(A) Cortical thickness differences of PD patients with MCI and healthy controls; (B) Cortical thickness differences of PD patients without MCI and healthy controls; (C) Cortical thickness differences of PD patients with MCI and PD patients without MCI. The scale bar shows t values, FDR correction, P < 0.05, size > 50.

SBM analysis revealed that PD patients with MCI had smaller cortical thicknesses in the temporal lobes (e.g., left middle temporal gyrus and right superior temporal gyrus) and the occipital cortex (e.g., left cuneus) compared to healthy controls as well as PD patients without MCI (Table 3 and Fig 1). Compared to the PD patient without MCI, the PD patients with MCI also showed cortical thinner in the left precuneus (Table 3 and Fig 2).

**Table 3:** Differences of cortical thickness between the groups by SBM analysis.

Comparisons	Brain regions	MNI Coordinate			t	Voxels
		x	y	z		
PD patients with MCI vs. healthy controls	L Middle Temporal Gyrus	-4	-89	19	-4.74	177
	R Superior Temporal Gyrus	6	-85	-26	-5.34	379
PD patients without MCI vs. healthy controls	L Cuneus	-9	-90	24	-3.51	278
PD patients with MCI vs. PD patients without MCI.	L Precuneus	-5	-66	40	-3.93	79

### 3.4 ROC analysis

**Fig 3:**
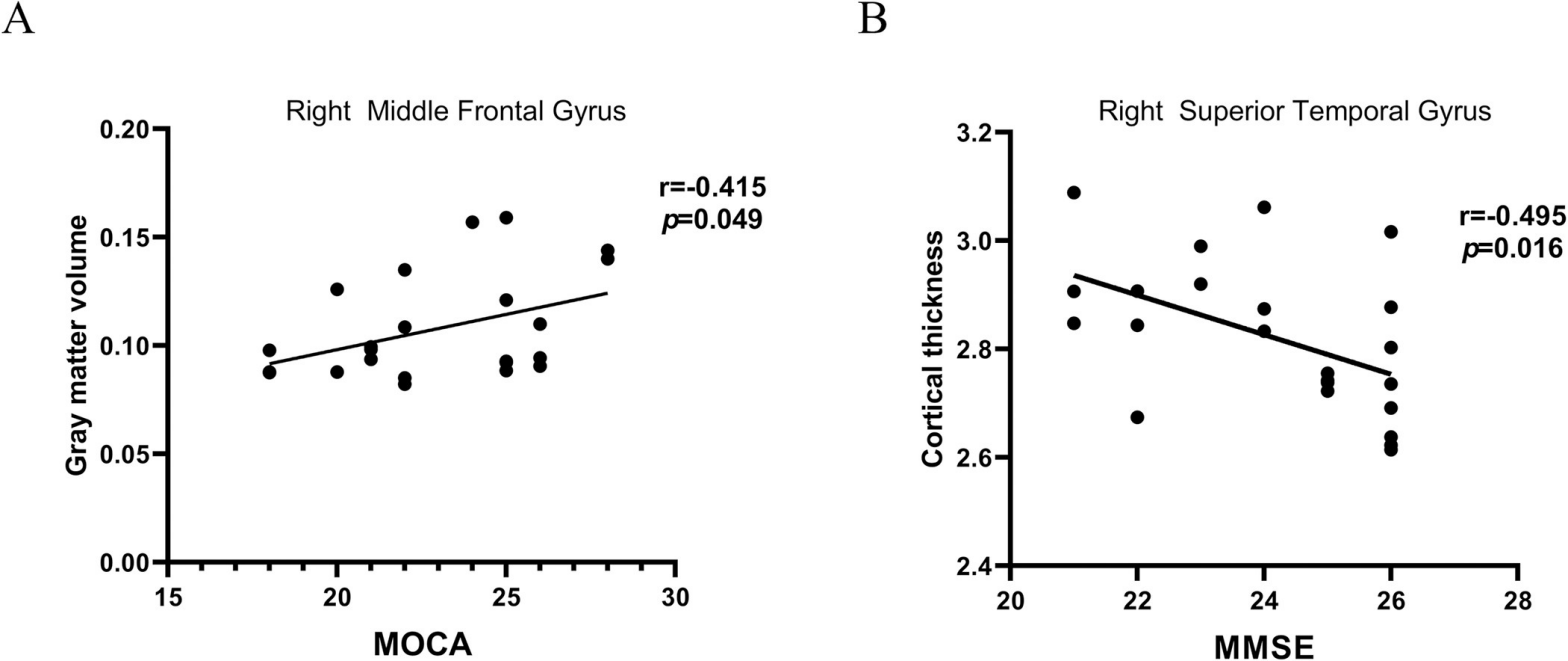
Receiver operating curve (ROC) analyses of the brain structure measurements in the gray matter volume (left column) and cortical thickness (right column) for differentiating PD with MCI from PD patients without MCI (A) and healthy controls (B). RMFG, right middle frontal gyrus; RIFG, right inferior frontal gyrus; RPUT, right putamen; LPUT, left putamen; RINS, right insula; RCER8, right cerebellum lobule VIII; LMTG, left middle temporal gyrus; RSTG, right superior temporal gyrus; LCUN, left cuneus; LPCUN, left precuneus. AUC, area under the ROC curve.

The results of ROC curve analysis showed that the AUC values of in the frontal (e.g., right middle frontal gyrus), temporal (e.g., left middle temporal gyrus), and subcortical structures (e.g., insula and cerebellum) were significant (>0.7) in detecting PD with MCI and PD without MCI patients and healthy controls (Fig 3).

### 3.5 Correlation analysis

**Fig 4:**
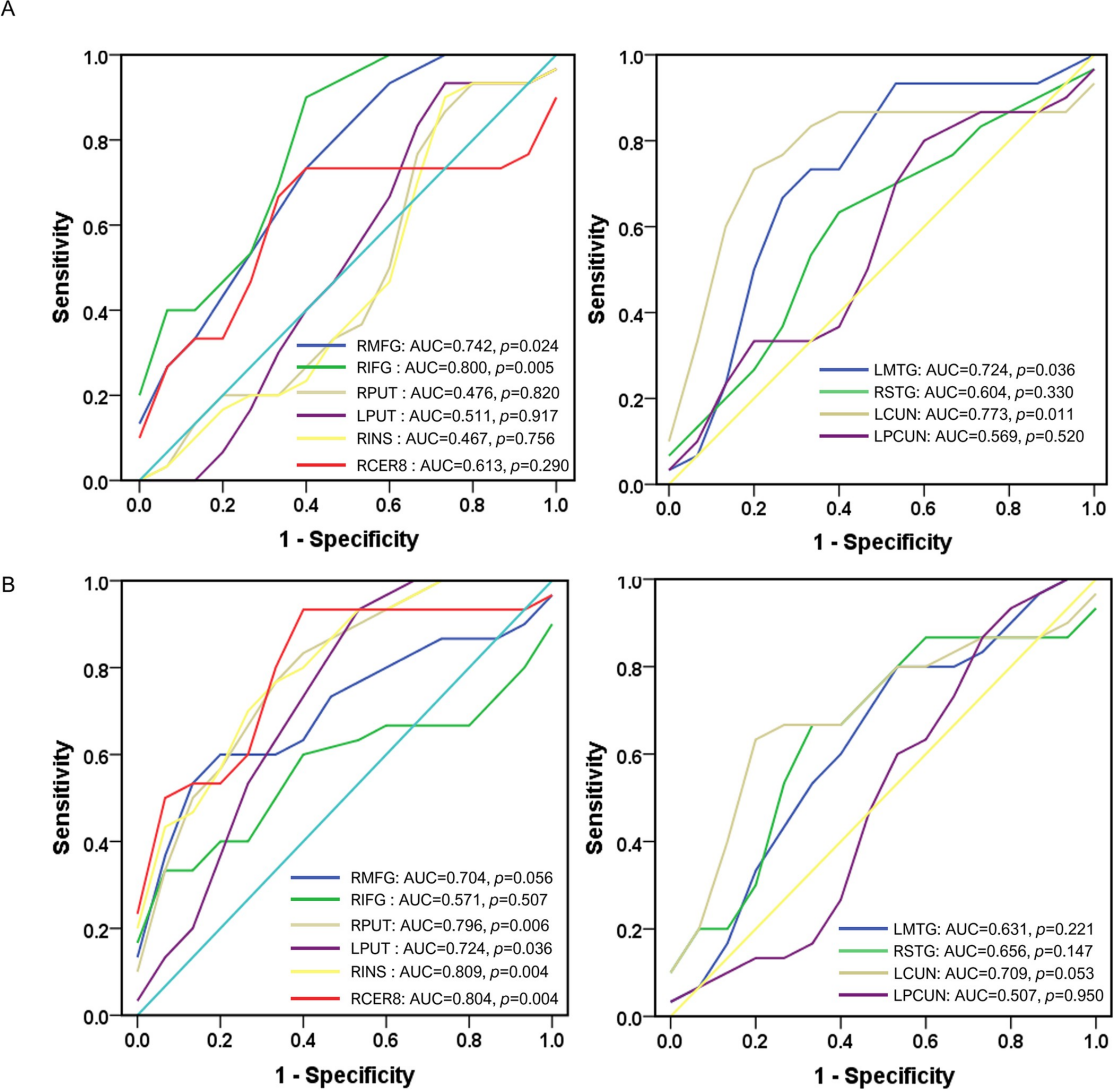
Correlations between morphometrics and neurocognitive scores in PD patients with MCI. (A) Gray matter volume of right middle frontal gyrus was positively correlated with MOCA scores; (B) Cortical thickness of right superior temporal gyrus was negatively correlated with MMSE scores. MFG, middle frontal gyrus; STG, superior temporal gyrus.

In the PD patient with MCI group, there was a positive correlation between GMV of the right middle frontal gyrus and MOCA score (r = 0.415, P = 0.049). The cortical thickness of the right superior temporal gyrus was negatively correlated with MMSE score (r = -0.495, P = 0.016). But no correlation was found in the other groups. The results are shown in Fig 4.

## 4. Discussion

The present study investigated the structural changes of the brain in PD patients with MCI by combing VBM and SBM analyses. The results showed that the PD patients with MCI had extensive atrophy (e.g., reduced GMV and cortical thickness) in different region, including the frontal and temporal lobes, but also subcortical structures (e.g., putamen, insula, and cerebellum) relative to PD patients without MCI and healthy controls. Interestingly, the discrimination of PD with MCI from PD without MCI patients and healthy controls showed promise in detecting the structural changes of the fronto-temporal regions and subcortical structures. And these abnormal structural changes were associated with neurocognitive functional declines in PD patients with MCI. These results provide relatively comprehensive aspects of brain pathological alternations in PD patients with MCI and initially support our hypothesis.

Structural neuroimaging helps investigate brain morphological features of PD patients with MCI. VBM analysis can detect subtle structural and neuropathological alterations in the whole brain and quantify alterations of GMV in PD [36]. Gray matter loss has been reported in cortical and subcortical regions in PD patients [37, 38]. We found that PD patients with MCI showed a more localized gray matter loss in the frontal cortex (e.g., middle frontal gyrus and inferior middle gyrus), parallel with previous studies [39–41]. The frontal regions involved in multiple important cognitive functions [42–44]. Gray matter abnormality in the frontal cortex was associated with impaired with executive function, attention, memory, and language abilities [45–47]. Therefore, it is worth noting that there was a significant correlation between gray matter atrophy of the frontal cortex (e.g., the middle frontal gyrus) and neurocognitive deficits (e.g., MOCA) in PD patient with MCI group, which indicates that lower GMV in the middle frontal gyrus suggests worse cognitive functions and a potential relationship between cognitive declines and structural loss in the brain region in PD.

We also found a decrease in gray matter signal in the frontal cortex extending to the insula and cerebellum (e.g., lobule VIII) in the PD patients with MCI. In addition to primary motor disorder, PD is well characterized by a number of non-motor symptoms rang from cognitive changes to sensory changes [48]. The insula is highly interconnected with the basal ganlia [49] and other cortical regions (e.g., the frontal, parietal, and temporal cortices) [50]. Thus, the insula can interact with multiple brain regions and plays a central role in directing cognitive processes [51]. Studies have revealed that reduced GMV of the insula was significantly correlated with cognitive dysfunction in PD patients with MCI [14, 52]. Imaging studies emphasize the distinct representation in the anterior (e.g., lobule IV-V) and posterior (e.g., lobule VIII) cerebellum, involved in motor control and cognitive function respectively [53, 54]. VBM analysis indicated that reduced GMV in the cerebellum was associated with cognitive impairment in PD patients [55]. Indeed, cognitive impairments together with the presence of motor deficits have been associated with atrophy of the cerebellum in PD patients [56]. The results implied that PD patients with MCI had grapy matter atrophy from cortical extending to subcortical areas and related with cognitive status, which could be underlying neural architecture that contributed to cognitive deficits.

The increased GMV in the putamen in the PD patients with NCI was unexpected but was consistent with the previous studies [57, 58], and may support the concept of striatum compensation in PD. The putamen is the striatal nucleus mainly linked with motor performance. The putamen and the motor areas are functional integrated during motor task performance [59]. This region had enhanced functional connectivity with motor cortex (e.g., supplementary motor area) [60, 61]. A disrupted pattern of the motor network could cause observable motor deficits in PD. The increased GMV in the putamen in PD patients with NCI may reflect a structural correlate of functional compensation for motor deficit, since higher volume of putamen was related better motor performance [62].

Moreover, the cortical gray matter layer covering the surface of the brain, referred to as cortical thickness, is valuable measurements to assess the neuroanatomical patterns associated with neurodegenerative diseases [63]. Neuroimaging studies has reported that PD patients with MCI had thinner cortical thickness in the temporal regions [63, 64]. In line with these findings, we found reduced cortical thickness in the temporal lobe (e.g., middle temporal gyrus and superior temporal gyrus) in PD patients with MCI. The temporal lobe plays an important role in cognitive functions [65]. Studies have demonstrated that there was a close correlation between the cortical thickness of temporal lobe and cognitive performance in PD patients [19, 66]. Therefore, we observed that the cortical thickness of the temporal lobe (e.g., superior temporal gyrus) was correlated with cognitive measurement (e.g., MMSE) of PD patients with MCI. The cortical thinning of precuneus was also found in the PD patients with MCI. The cortical atrophy of the precuneus seems to contribute to cognitive declines in PD [67]. The results support previous studies in which cognitive impairments in PD related to temporo-parietal surface area [68, 69]. In addition, the occipital cortex (e.g., cuneus) implicated in PD patients with NCI showed relative decreased cortical thickness. The result was line with the previous studies [70, 71]. The cuneus is one of the earliest regions to show cortical atrophy [72] and was associated with motor symptoms in PD [70]. These results suggested that there was evidence of widespread cortical brain changes in PD with MCI and may resulted in a series of clinical manifestations in the disease.

Interesting, the altered brain structure, including the cortical regions (e.g., the frontal, and temporal lobes) and subcortical structures (e.g., the insula and cerebellum), were sensitive for differentiating the patients from different subtypes and healthy controls. Atrophy in the frontotemporal areas was found in patients with MCI compared to healthy controls [20, 39], and cognitive impairments are associated with gray matter loss in the areas [73]. Gray matter atrophy of cortical structures extending to the subcortical areas (e.g., insula and cerebellum) presents in the PD patients with MCI [11, 74]. The subcortical structural deficits are functionally related to widespread cognitive functions [75, 76]. The results confirmed that MCI was associated with widespread brain atrophy and indicated that the decreased gray matter volume or cortical thickness in these brain structures could be biomarkers for cognitive impairments in PD.

There were several limitations in the present study that need to be pointed out. First, the sample size of the current study was relatively small and could affect the statistical power of the results. A larger sample size is needed to validate the analysis. Second, the patients in the current sample were already taking anti-parkinsonian medication, the dopaminergic treatment may affect MRI measurements. Studying early stage and untreated PD patients using MRI could gain better understanding of the disease regardless of pharmacological treatment. Finally, this study focused only on gray matter. Combining gray matter and white matter features are key to provide a fuller framework for the underlying pathological processes in the PD population.

## 5. Conclusion

Both VBM and SBM methods provided complementary information on neurodegenerative changes in PD patients with MCI. PD patients with MCI had widespread structural alternations and these structural abnormalities may be associated with the pathophysiological basis of PD and could be biomarkers of MCI in PD.

## Supporting information

S1 Data (XLSX)
